# A global comparison of the microbiome compositions of three gut locations in commercial pigs with extreme feed conversion ratios

**DOI:** 10.1038/s41598-018-22692-0

**Published:** 2018-03-14

**Authors:** Jianping Quan, Gengyuan Cai, Jian Ye, Ming Yang, Rongrong Ding, Xingwang Wang, Enqin Zheng, Disheng Fu, Shaoyun Li, Shenping Zhou, Dewu Liu, Jie Yang, Zhenfang Wu

**Affiliations:** 1 College of Animal Science and National Engineering Research Center for Breeding Swine Industry, South China Agricultural University, Guangdong, P.R. China; 2 National Engineering Research Center for Breeding Swine Industry, Guangdong Wens Foodstuffs Co., Ltd, Guangdong, P.R. China

## Abstract

In an attempt to increase profits and sustainability in the swine industry, the gut microbiome has become a focus of much research. In this study, we performed a comparative analysis of the gut microbiome in the ileum, cecum, and colon of Duroc × (Landrace × Yorkshire) (DLY) pigs showing two extreme feed conversion ratios (FCRs) using 16S rRNA gene sequencing. The results revealed that the microbial community in the cecum and colon had significantly higher alpha diversity than the ileum. We further identified 11, 55, and 55 operational taxonomic units (OTUs) with significantly different relative abundances between the high and low FCR pigs among the three gut locations, respectively. These OTUs were mainly associated with bacteria that participate in the metabolism of dietary polysaccharides and proteins. We then identified two and nine metabolic pathways that were enriched in the cecum and colon of the high FCR pigs, respectively. The results suggested that the short chain fatty acids and indolic compounds produced by microbial fermentation might influence porcine feed efficiency. These results should improve our understanding of microbiota compositions in the different gut locations of commercial pigs and provide important insights into the effect of gut microbiota on porcine FCRs.

## Introduction

Pork is a major meat source for humans, and the Duroc × (Landrace × Yorkshire) (DLY) cross is the most widely bred pig in the swine industry. In swine production, the feed conversion ratio (FCR) is an important economic metric that is used to measure feed efficiency. Low FCR values result in relatively high pork prices as a result of elevated feeding costs. Increasing the FCR of commercial pigs thus represents an important strategy for minimizing swine production costs.

Previous studies have revealed that many factors influence feed efficiency, including animal genetics, diet, disease, and production management^1–3^. In addition to these factors, increasingly studies have demonstrated that the intestinal microbiota in pigs play a crucial role in nutrient processing and energy harvesting^4,5^, which, in turn, leads to variation in feed efficiency. Ramayo-Caldas *et al*. showed that porcine gut microbial composition was significantly associated with body weight and average daily gain^6^. Moreover, many studies have also shown that intestinal microbiota can provide an extensive array of enzymes and substrates for the host, resulting in improved host energy and nutrient supply^7,8^. For example, Turnbaugh *et al*. and Looft *et al*. discovered that many metabolic enzymes encoded by microbial genomes can degrade plant cell wall components that otherwise cannot be digested by the host into short chain fatty acids (SCFAs) in the swine intestine^9,10^. Delzenne *et al*. identified numerous microbial genes related to the synthesis of essential amino acids and vitamins in the porcine large intestine^11^.

Furthermore, Yang *et al*. reported the relationship between fecal microbiota with feed efficiency in Duroc pigs^12^. Tan *et al*. detailed the relationship between cecal microbiota and feed efficiency in Landrace pigs^13^, while McCormack *et al*. analyzed the association of ileum and cecal microbiota with feed efficiency in Large White × Landrace pigs^14^. However, to our knowledge, few studies have analyzed the association between the microbiotal composition and functional capacity of the ileum, cecum, and colon with regards to feed efficiency in DLY pigs.

Pigs are recognized as important animal models in gastrointestinal tract studies due to their nutritional similarities with humans in terms of nutrient absorption and utilization^15^. Generally, pigs with a high FCR are capable of obtaining more energy and nutrients, thus gaining more weight, from the same amount of feed than pigs with a low FCR^16^. However, pigs with higher caloric gain will be more likely to deposit body fat, especially when the caloric gain from the feed exceeds caloric utilization^17^. Consequently, given the same quantity of feed intake, high FCR pigs are also prone to becoming obese^17^, which can be linked to non-genetic diseases associated with obesity in humans. Many previous studies that investigated the gut microbiotal composition of pigs were performed using feces^12,18,19^. The microbial community in the fecal matter is likely to represent only a discrete population of the intestinal microbiota, rather than the total composition of the gut microbiome. Conversely, the direct sampling of specific gut locations has been shown to provide information relating to the differential composition of gut microbial communities^9^.

The 16S rRNA gene is a phylogenetic marker that can be used to classify microbial taxa, allowing for the analysis of microbial community composition in intestinal content samples^20^. In the present study, we performed 16S rRNA gene sequencing analysis of the gut microbiome at three different gut locations in 12 pigs with high FCRs and 12 pigs with low FCRs. Our aim was to characterize the differences in gut microbiome composition between the high and low FCR pigs and evaluate the underlying association of the gut microbiota with feed efficiency.

## Results

### Metadata and sequencing

Based on the weight data and feed intake recordings, the FCRs differed significantly between the high and low FCR groups (2.23 ± 0.07 *vs*. 2.65 ± 0.07, *P = *7.4e−07) on day 140 (Supplementary Fig. S1A). A total of 72 luminal samples (high FCR groups: 12 ileum samples, 12 cecum samples, 12 colon samples; low FCR groups: 12 ileum samples, 12 cecum samples, 12 colon samples) were collected and sent for high-throughput sequencing. A total of 5,327,446 sequencing reads were generated and clustered into 4,154,585 tags. Following OTU selection and chimera checking, all non-unique tags were assigned to 58,426 OTUs, with an average of 811 OTUs per sample. Both of Good’s coverage indices in the ileum, cecum, and colon were greater than 99%, indicating sufficient data sampling and adequate sequencing depth (Supplementary Fig. S1B).

### Difference in swine intestinal bacterial community composition between the three gut locations

**Figure 1:**
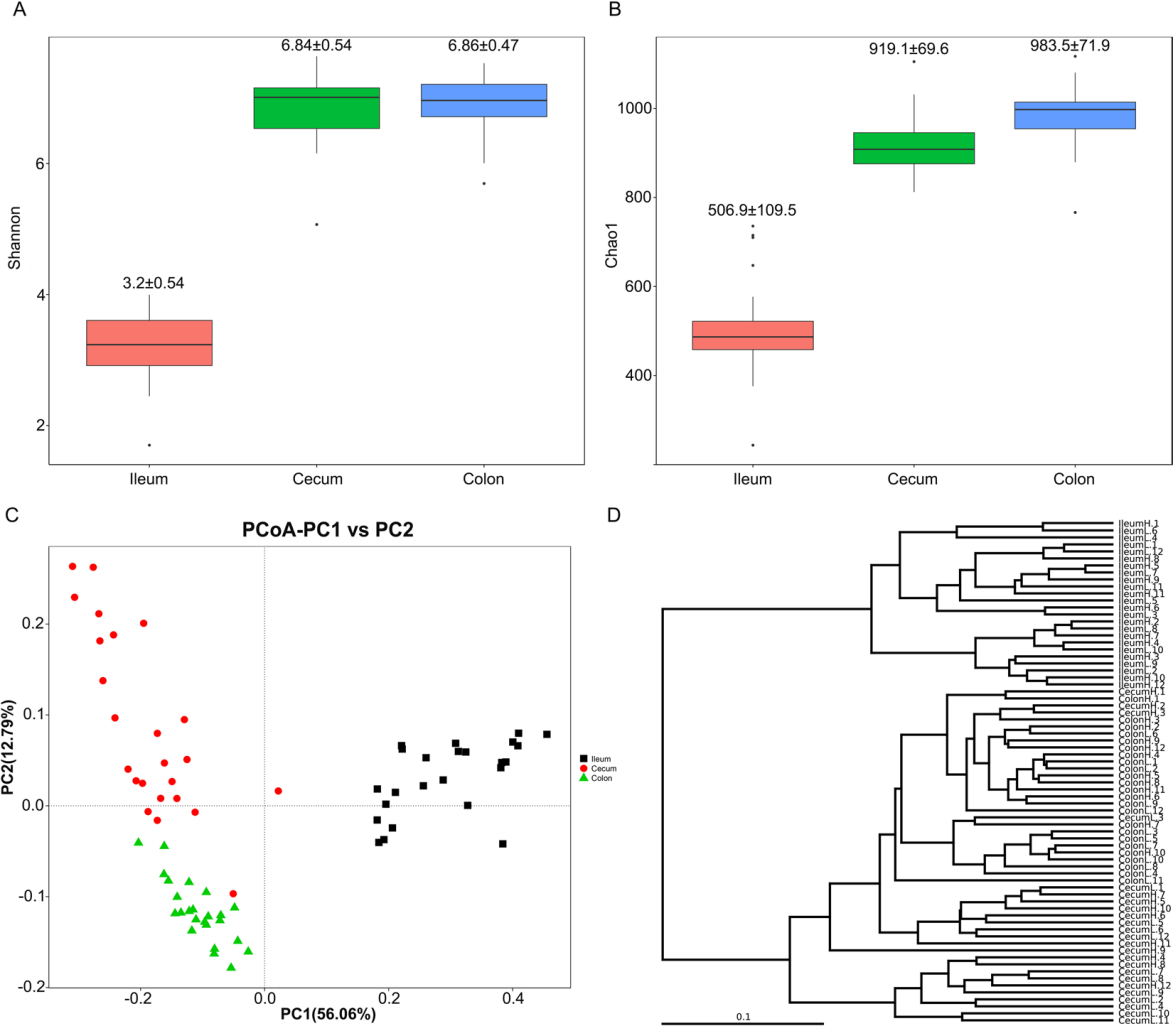
The alpha- and beta- diversity comparisons for the ileum, cecum, and colon. (**A**) The Shannon’s diversity index at the sampling site (mean ± SD). (**B**) The Chao1 index at the sampling site (mean ± SD). (**C**) Weighted UniFrac PCoA of the microbiota. Each symbol and color represents each gut location microbiota. (**D**) Bray Curtis dendrogram analyses were performed on the 16S rRNA V4–V5 region data. Samples are coded to represent the sampling site (Ileum; Cecum; Colon) and feed conversion ratio status (H, high feed conversion ratio; L, low feed conversion ratio). For example, IleumH represented a sample that was collected from the ileum of a high FCR pig.

To evaluate the phylogenetic composition of the bacterial communities in the different gut locations, we first performed OTU analysis and compared the alpha diversity of the microbiota from the three intestinal regions. The average number of OTUs differed significantly among the three gut locations, with incremental increases in each subsequent gut section: ileum < cecum (522.8 ± 98.7 *vs*. 916.8 ± 65.7, *P* < 0.01), cecum < colon (916.8 ± 65.7 *vs*. 994.8 ± 98.7, *P* < 0.01) (Supplementary Fig. S1C). The Shannon index, which measures species richness and evenness, and the Chao1 index, which estimates species richness, were calculated in order to thoroughly evaluate the alpha diversity. The ileum was associated with significantly lower Shannon and Chao1 indices than the cecum and colon (*P* < 0.01). Additionally, the colon had a significantly higher Chao1 index than the cecum (*P* < 0.05). However, no significant differences in the Shannon index were observed between the cecum and colon (Fig. 1A and B).

We subsequently explored the variations in microbial community composition and the degree of similarity between the samples from the three gut locations at the OTU level. A Principal Coordinate Analysis (PCoA) plot, based on the weighed Unifrac distance matrices, showed that the gut bacteria composition differed significantly at the different gut locations. The microbiotal composition in the ileum samples was significantly different from that of the cecum and colon, while the cecum and colon samples were more similar to each other (Fig. 1C). Furthermore, Bray Curtis clustering analysis of gut bacteria at the OTU level showed that most samples from the three gut locations could be clustered into three subgroups, with the exception of three cecum samples (CecumH.1, CecumH.2, and CecumH.3) in the high FCR group, and one cecum sample in the low FCR group (CecumL.3). These exceptions clustered with the colon samples in the dendrogram (Fig. 1D). These results were consistent with the multiple response permutation procedure (MRPP) analysis, which suggested that the microbial community structure of the three gut locations differed significantly, but the difference between the cecum and colon samples was smaller than that between the ileum and cecum or colon (Supplementary Table S1).

**Figure 2:**
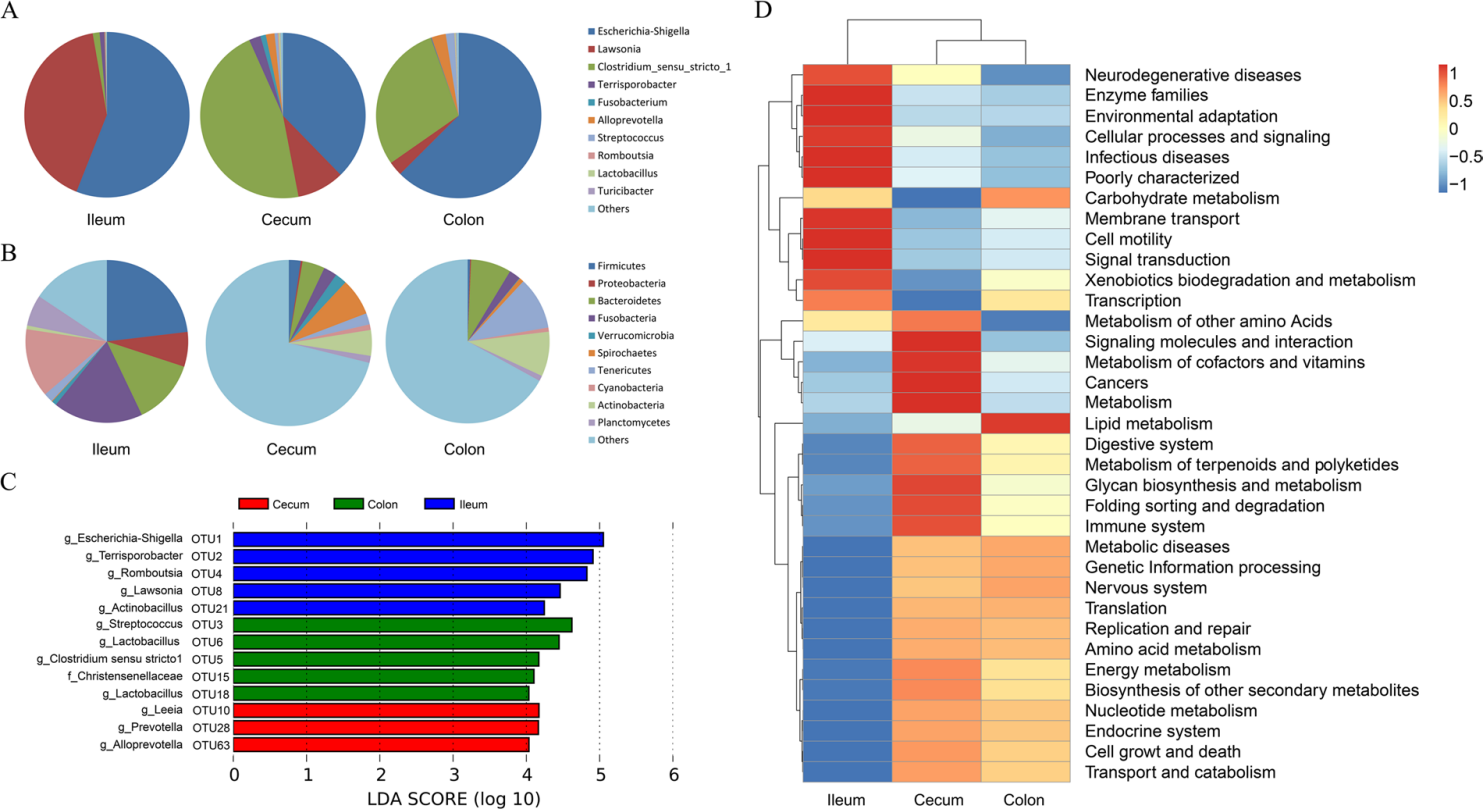
Relative abundance analyses at the OTU level and functional capacity profiles of the microbial communities of the ileum, cecum, and colon derived from 16S rRNA gene sequencing data. (**A**) Microbial composition at the phylum level. (**B**) Microbial composition at the genus level. Gut locations are represented along the horizontal axis, and relative abundance is denoted by the pie charts. (**C**) LEfSe identified significantly different OTUs according to the relative abundance among the three gut locations. The histogram shows the LDA scores computed for features at the OTU level, and each OTU was annotated at the lowest classification level (p: phylum, c: class, o: order, f: family, g: genus) according to the GreenGenes Database. OTUs in this graph were statistically significant (*P* < 0.05) and had an LDA Score >±4.0, which was considered a significant effect size. (**D**) Predicted function of the gut microbiota in the ileum, cecum, and colon. The vertical columns represent groups, and the horizontal rows depict metabolic pathways. The color coding is based on row z-scores.

Furthermore, we explored the taxonomic distribution of the numerically abundant bacteria in each gut location. Based on the bacterial relative abundance of the top 10 phyla, Firmicutes constituted the most prevalent phylotype, comprising 56.0% of the ileum microbial population, followed by Proteobacteria, which occupied 41.2% of the microbial population. However, Proteobacteria only accounted for 9.27% and 2.78% of the relative abundances in the cecum and colon, respectively. The relative abundance of Bacteroidetes was 1.34%, 46.4%, and 29.2% in the ileum, cecum, and colon, respectively. In addition, we also observed Fusobacteria (2.28%) and Verrucomicrobia (1.06%) in the cecum, and Spirochaetes (2.80%) in the colon (Fig. 2A and Supplementary Table S2). At the genus level, *Escherichia-Shigella* (23.1%), *Terrisporobacter* (17.9%), *Romboutsia* (13.7%), and *Clostridium sensustricto1* (12.9%) were most prevalent in the ileum. *Alloprevotella* (7.2%), *Lactobacillus* (5.0%), and *Prevotellaceae NK3B31group* (4.4%) were the three most prevalent genera in the cecum. *Streptococcus* (10.4%), *Lactobacillus* (8.8%), and *Clostridium* (8.0%) were the three most prevalent genera in the colon (Fig. 2B and Supplementary Table S3). These results indicated a greater relative abundance of bacteria in the ileum mainly consisted of several genera, while the bacteria in the cecum were more uniformly distributed.

In order to identify specific bacterial species that were characteristic to the three gut locations, we performed linear discriminant analysis coupled with effect size (LEfSe) on the taxa that exhibited linear discriminant analysis (LDA) scores greater than four. Figure 2C indicates the 13 OTUs that were differentially represented among the three gut locations. The relative abundance of the five OTUs was higher in the microbiome community of the ileum than in the cecum and colon. These OTUs were represented by the genera *Escherichia-Shigella*, *Terrisporobacter*, *Romboutsia*, *Lawsonia*, and *Actinobacillus*. Three OTUs, including OTU10 (*Leeia*), OTU28 (*Prevotella*) and OTU63 (*Alloprevotella*) were relatively more abundant in the cecum than in the other two gut locations. In the colon, five other OTUs exhibited higher relative abundance compared to the other gut locations, and two of these OTUs were represented by the genus *Lactobacillus* (Fig. 2C and Supplementary Table S4).

### Comparison of the functional capacity of the microbiota among the three gut locations

PICRUSt was used to evaluate the functional profiles of the pig gut microbiota in the three gut locations and to explore the potential links with FCR^21^. The results indicated that 11 pathways, including neurodegenerative diseases, enzyme families, environmental adaptation, cellular processes and signaling, infectious diseases, poorly characterized, membrane transport, cell motility, signal transduction, xenobiotics biodegradation and metabolism, and transcription were overrepresented in the ileum in comparison to the cecum and colon. Ten pathways, including metabolism of other amino acids, signaling molecules and interaction, metabolism of cofactors and vitamins, cancers, metabolism, digestive system, metabolism of terpenoids and polyketides, glycan biosynthesis and metabolism, folding sorting and degradation, and immune system were overrepresented in the cecum than the ileum and colon. Two pathways, including carbohydrate metabolism and lipid metabolism, were overrepresented in the colon than the ileum and cecum. In summary, the most important predicted functional capacities of the intestinal microbiota were enzymatic digestion in the ileum, glycan biosynthesis, and multi-metabolic capabilities, which included protein metabolism, vitamin metabolism, terpenoid metabolism and polyketide metabolism in the cecum, and lipid and carbohydrate fermentation in the colon (Fig. 2D and Supplementary Table S5).

### Comparison of the microbial community structure in the different intestinal locations of pigs from the different FCR groups

**Figure 3:**
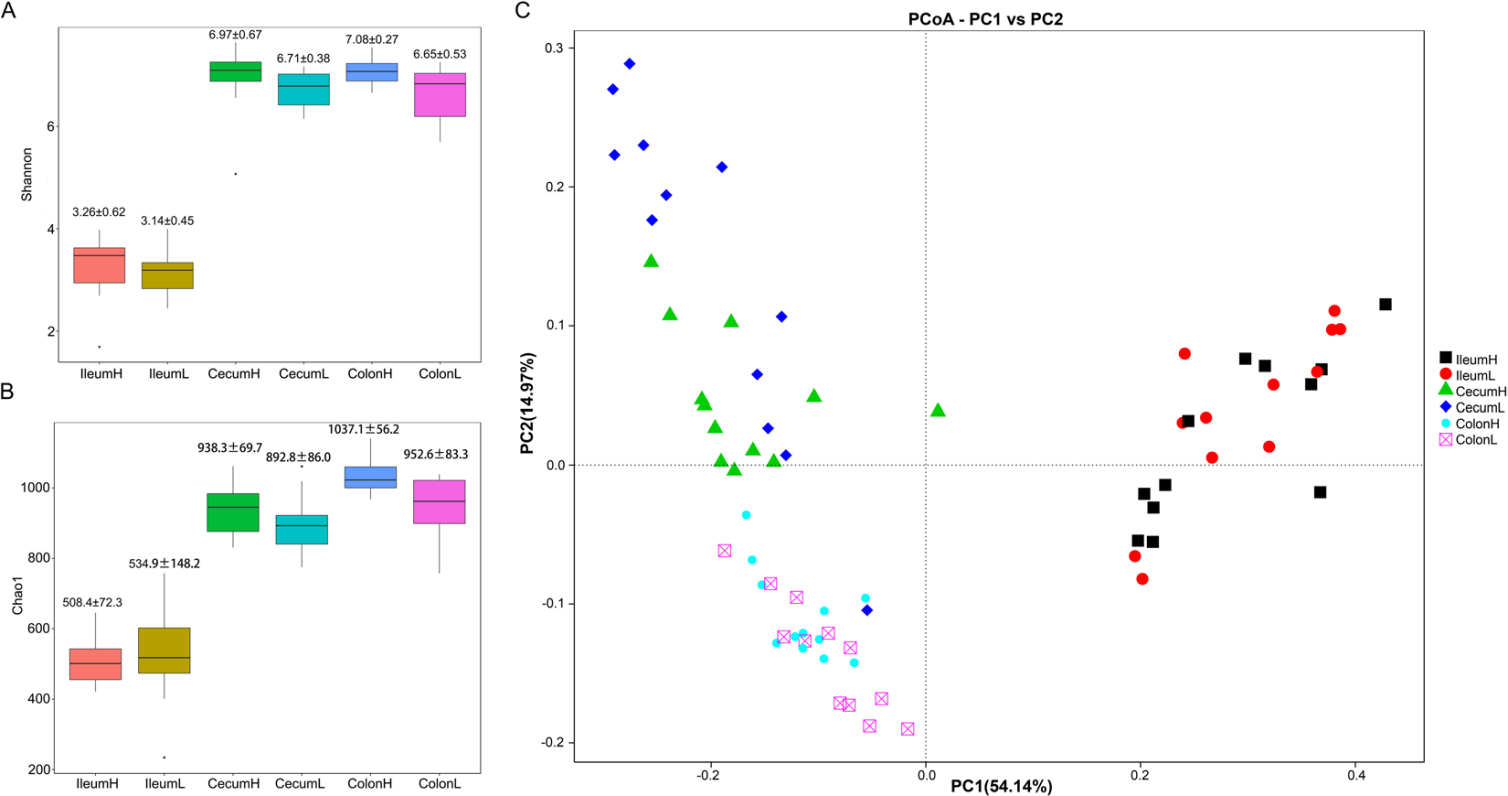
The alpha- and beta- diversity comparisons of high and low FCR groups in each gut location. (**A**) Shannon’s diversity index between high and low FCR pigs (mean ± SD). (**B**) The Chao1 index between high and low FCR pigs (mean ± SD). (**C**) Weighted UniFrac PCoA of the microbiota from distinct FCR groups.

Gut luminal samples for 16S rRNA gene sequencing were collected from pigs with high and low FCRs. The alpha diversities between the pigs with different FCRs in each gut location (ileum, colon, and cecum) were then compared. A significantly higher Shannon index was observed in the high FCR pigs compared with the low FCR pigs in the cecum and colon (*P* < 0.05, Fig. 3A). The high FCR group had a significantly higher Chao1 index than the low FCR group in the colon (*P* < 0.01, Fig. 3B); however, the Shannon and Chao1 indices in the ileum samples were not significantly different between the high and low FCR pigs. A PCoA plot, based on weighted Unifrac, did not show distinct differences in microbial structures between high and low FCR pigs (Fig. 3C).

**Figure 4:**
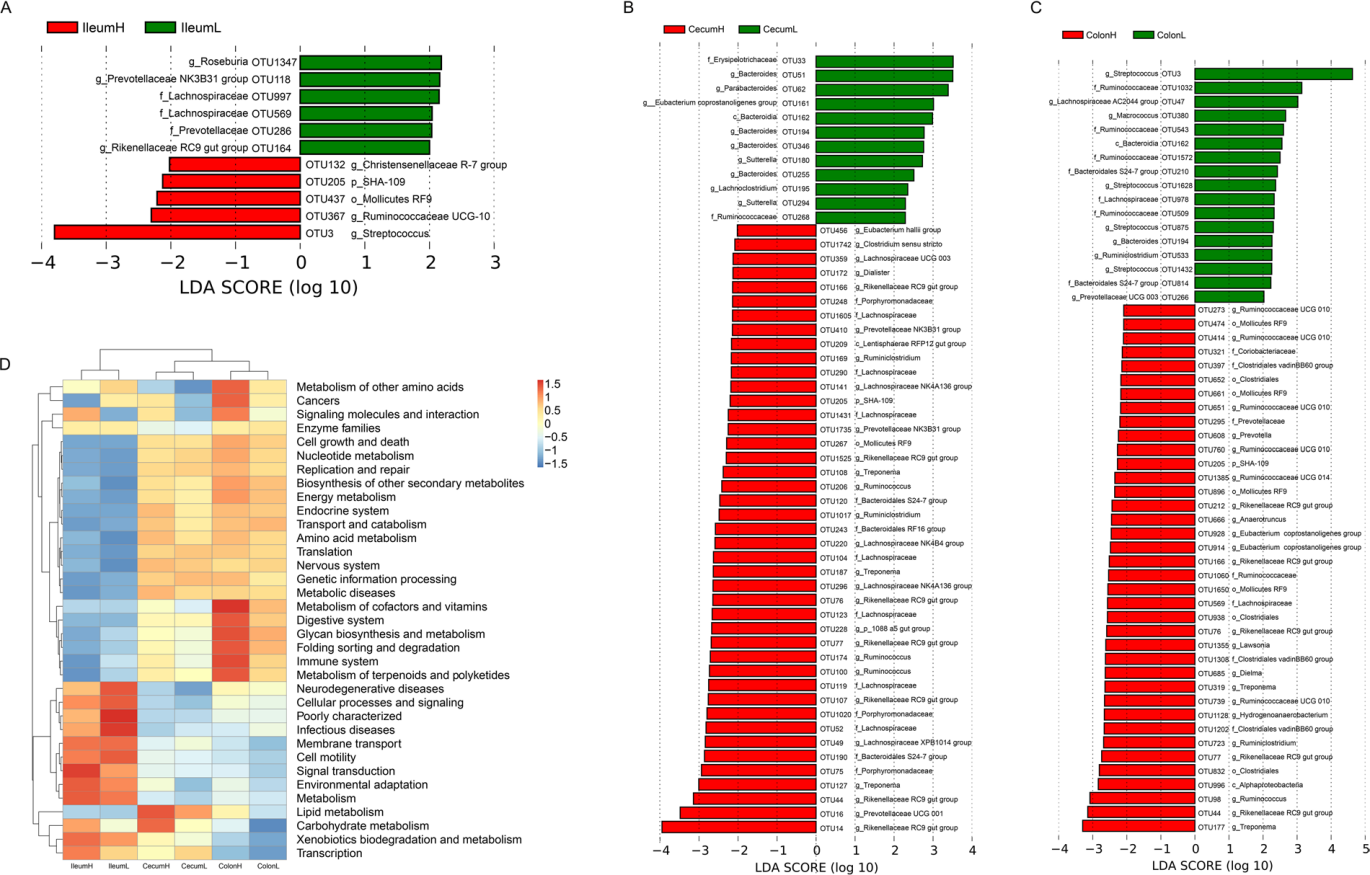
Taxonomic and functional capacity profiles of the microbial communities of high and low FCR groups in each gut location. LEfSe identified significantly different bacterial taxa (at OTU level) between the high and low FCR groups in the ileum (**A**), cecum (**B**), and colon (**C**). OTUs in this graph were statistically significant (*P* < 0.05) and had an LDA Score >±2.0, which was considered a significant effect size. (**D**) Predicted function of the gut microbiota between high and low FCR groups at each gut location.

Supplementary Figs S2A and S2B show the community composition of the high and low FCR pigs in the three gut locations at the phylum and genus level, respectively. To identify specific bacterial species that were characteristic to the high and low FCR pigs, we performed LEfSe on the taxa with LDA scores greater than two. The results revealed that five OTUs were higher and six OTUs were lower in the ileum of the high FCR pigs than in low FCR pigs (Fig. 4A and Supplementary Table S6). In the cecum, 43 OTUs were higher and 12 OTUs were lower in high FCR pigs than in the low FCR pigs (Fig. 4B and Supplementary Table S6). In the colon, 38 OTUs were higher and 17 OTUs were lower in high FCR pigs than in low FCR pigs (Fig. 4C and Supplementary Table S6). In all three gut locations, OTU205 (*SHA-109*) showed significantly higher relative abundance in high FCR pigs than in the low FCR pigs (Fig. 4). In the ileum, OTU3 (*Streptococcus*), OTU132 (*Christensenellaceae R-7 group*), and OTU367 (*Ruminococcaceae UCG-010*) were dominant in the high FCR pigs, while OTU118 (*Prevotellaceae NK3B31 group*), OUT164 (*Rikenellaceae RC9 gut group*), and OTU1347 (*Roseburia*) were dominant in the low FCR pigs (Fig. 4A). In the cecum, 12 OTUs that represented the family Lachnospiraceae were more abundant in the high FCR pigs. The high FCR pigs were enriched in three OTUs that represented the genus *Ruminococcus* and two OTUs that represented the genus *Ruminiclostridium*, which both belong to the Ruminococcaceae family. Moreover, the high FCR pigs were enriched in seven and three OTUs that represented the families of Rikenellaceae and Prevotellaceae, respectively (Fig. 4B). In the colon, nine and five OTUs that respectively represented the families of Ruminococcaceae and Rikenellaceae were more abundant in the high FCR pigs. Furthermore, the high FCR pigs possessed more OTUs that represented the genera *Hydrogenoanaerobacterium* and *Anaerotruncus* (Fig. 4C).

Similarly, we used PICRUSt to explore the different metabolic pathways of the ileum, cecum, and colon microbiome in high and low FCR pigs. In the ileum, the pathways of neurodegenerative diseases, poorly characterized, and infectious diseases were more enriched in the low FCR group compared with the high FCR group. The pathways of carbohydrate metabolism and lipid metabolism were more enriched in the cecum of the high FCR group compared with the low FCR group. A total of nine pathways, including metabolism of other amino acids, cancers, signaling molecules and interaction, metabolism of cofactors and vitamins, digestive system, glycan biosynthesis and metabolism, folding sorting and degradation, immune system, and metabolism of terpenoids and polyketides were more abundant in the colon of the high FCR group than in low FCR groups (Fig. 4D and Supplementary Table S7). This suggests that the improvement in FCR in pigs may be affected by the enhanced functional capacities of the microbiota in the cecum and/or colon.

## Discussion

High-throughput sequencing methods provide a more direct way to analyze microbial taxa in comparison to culture-dependent methods, especially when dealing with fastidious species^22^. Many studies have thus focused on high-throughput sequencing methods to explore the gut microbiome composition of animals^23–28^. However, most previous studies used fecal samples to investigate the gut microbiome composition of pigs^12,18,19^. It is well-known that fecal bacteria are composed of discrete downstream populations that may not be representative of the entire intestinal bacterial community. Therefore, studying the bacteria obtained from different gut locations can improve our ability to make inferences about coevolved bacterial partners in complex mammalian ecosystems^9^. In this study, we compared the microbial community structure and microbiome composition from the anterior and posterior gut in different FCR groups using 16S rRNA gene sequencing. To the best of our knowledge, this is the first report to analyze the effect of different FCRs on the gut microbial community and functional capacity in the swine ileum, cecum, and colon. Furthermore, no previous studies have evaluated the relationship between gut microbiota and feed efficiency in the DLY hybrid population, which constitutes the largest commercial pig variety worldwide. The present study addresses this and also offers important insights into the role of gut microbiota in the FCR of commercial pigs.

Consistent with previous reports^9,29^, Firmicutes and Proteobacteria were the two most dominant phyla in the ileum. Unlike previous studies^9^, we found significant differences between the bacterial composition of the cecum and colon at the phylum level. At the lower taxonomic level, previous studies using 300-day-old Laiwu pigs revealed that *Clostridium* and *Prevotella* were the most prevalent genera in the ileum and cecum, respectively^30^. In another study, 90-day-old pigs showed a highly variable distribution of *Prevotella*, *Oscillibacter*, and *Succinivibrio* in the colon^9^. The prevalent genera in these studies differed substantially from our study. This may be attributed to the differences in the breed, age, feed, and husbandry of the analyzed pigs. Additionally, in our study, the microbial community compositions varied across the different gut locations. Previous studies revealed that many members of *Escherichia-Shigella* and *Romboutsia*, which are most abundant in the ileum, contributed to the degradation of glucose and fructo-oligosaccharides^31,32^. High populations of *Prevotella* and *Actinobacillus* in the cecum were previously associated with polysaccharide fermentation^33,34^. Many members of *Lactobacillus* and *Streptococcus* (the prevalent genera in the colon) contribute to lactic acid production^35^. Another study suggested that the enzymatic digestion and absorption of starch constitute the predominant functions of the small intestine, while the large intestine mainly functions to ferment non-starch polysaccharides via bacteria and produce SCFAs^36^, which serve as important nutrients for the epithelium and body tissues. Therefore, the functional capacities of the prevalent bacteria were quite similar to the physiological functions of the pig intestine. This suggests that in each gut location, the prevalent bacteria play an important role in the physiological functions of the particular location. In the ileum of the high FCR pigs, most of the dominant OTUs belonged to *Streptococcus*, Ruminococcaceae, and Christensenellaceae. The OTUs associated with *Streptococcus* were linked to lactic acid-producing bacteria^35^, and the OTUs associated with Ruminococcaceae were capable of producing SCFAs by fermenting dietary polysaccharides^37^. The OTUs associated with Christensenellaceae can produce volatile fatty acids as the end products of fermentation^38^. Moreover, previous studies revealed that the phyla of *SHA-109* were associated with carbohydrate degradation^39,40^. Interestingly, the ileum of the low FCR pigs contained more OTUs that were associated with the Lachnospiraceae, Prevotellaceae, and Rikenellaceae families. Many members of Lachnospiraceae are capable of producing butyric acid through the fermentation of various substrates^41^. Prevotellaceae and the Rikenellaceae can facilitate the breakdown of proteins and carbohydrates in feed^42,43^. The dominant OTUs in the ileum of the high and low FCR pigs have similar metabolic functions, and these results were consistent with the result of the PICRUSt analysis. The experimental pigs in this study were fed a formula diet that included fiber-enriched corn and high-protein soybean. Therefore, we hypothesized that the cecum or colon microbiome of the high FCR pigs might have a greater ability to utilize the crude protein or dietary indigestible cellulose. The most dominant OTUs in the cecum belonged to the Rikenellaceae, Prevotellaceae, Lachnospiraceae, and Ruminococcaceae families. Many members of these families show a high potential for fermenting various polysaccharides and dietary proteins^41–45^. Excluding the four families mentioned above, the colon had a greater abundance of OTUs that represented the genera *Anaerotruncus* and *Hydrogenoanaerobacterium*, which are involved in sugar fermentation with the concomitant production of formate and acetate as major metabolites^46^. Consistent with the results of the PICRUSt analysis, the Kyoto Encyclopedia of Genes and Genomes (KEGG) pathways related to carbohydrate and lipid metabolism were enriched in the cecum of the high FCR pigs, and the pathways related to protein metabolism were enriched in the colon of the low FCR pigs. Previous studies suggested that the fermentation of dietary polysaccharides could result in the production of SCFAs, which can improve the absorptive capacity of the intestine and increase porcine feed efficiency^12,47^. Gut microbiota are able to degrade proteins into diverse metabolites, including indolic compounds that maintain the epithelial barrier function of the intestinal tract^48^. However, the direct relationship between indole compounds and animal growth requires further investigation. Upon analysis of the microbiotal abundance, variability, and metabolic pathways in the different FCR pigs, we discovered that high FCR pigs generally exhibited a higher abundance of microbes that are beneficial for feed digestion and fermentation than low FCR pigs. Moreover, microbial metabolites, such as SCFAs, can improve the feed efficiency of swine.

## Conclusion

We detected 11, 55, and 55 OTUs that were potentially associated with swine FCR in the ileum, cecum, and colon, respectively. Our results suggested that the OTUs in the cecum and colon of the high FCR pigs might have a greater ability to utilize dietary polysaccharides and dietary protein compared to low FCR pigs, and the SCFAs and indolic compounds produced by microbial fermentation might improve porcine feed efficiency and promote intestinal health. However, the exact mechanisms remain unclear, thus warranting further investigation. These findings should improve our understanding of the microbiotal compositions in the different gut locations of commercial pigs and provide important insights into the effect of gut microbiota on porcine FCRs.

## Materials and Methods

### Statement of ethics for the care and use of animals

The experimental procedures used in this study met the guidelines of the Animal Care and Use Committee of the South China Agricultural University (SCAU) (Guangzhou, People’s Republic of China). All animal experiments in this study were approved by the Animal Care and Use Committee (ACUC) of the SCAU (approval number SCAU#0017).

### Animals and gut luminal sample collection

A total of 226 normal weaning (28-day-old) female DLY pigs were raised in a fattening house comprised of 30 pens, each housing 6–8 pigs. All pigs in this study were selected from populations with similar genetic backgrounds and husbandry practices. All of the pigs were fed twice a day with customized corn-soybean feed (free of probiotics and antibiotics) containing 16% crude protein, 3100 kJ of digestible energy, and 0.78% lysine. Water was available *ad libitum* from nipple drinkers. All animals were healthy and did not receive any antibiotic treatment in the two months prior to slaughter. The feed intake and body weight of each pig was measured by Osborne’s FIRE (Feed Intake Recording Equipment) System daily. All pigs were sorted according to their FCR value (feed intake divided by the weight gained) at 140 days of age. The 12 pigs with the highest FCR and the 12 pigs with the lowest were selected for this study. The selected pigs were slaughtered at 141–142 days of age, after overnight fasting. The analyzed pigs were from different pens, and the luminal content samples were collected at the same site for each gut location. Briefly, the luminal contents were separately gathered from the middle section of the colon and the ileum. Cecum luminal samples were collected from the bottom section of the cecum. The experimental platform was disinfected before each sample was collected to avoid cross-contamination between samples. All samples were harvested within 30 min of slaughtering and transferred immediately to liquid nitrogen for temporary storage. Samples were then sent to the laboratory where they were stored at −80 °C until analysis.

### DNA extraction, PCR amplification, and sequencing

Total bacterial DNA from the luminal samples was extracted using a Soil Genome^TM^ DNA Isolation kit (Qiagen, Germany), according to the manufacturer instructions. DNA concentration and quality were measured using UV-Vis spectrophotometry (NanoDrop 2000, USA) and agarose gel electrophoresis. The DNA obtained from each sample was diluted to 1 ng/μL with sterile water. Amplification of the V4–V5 hypervariable region of the bacterial 16S rRNA gene was performed using universal primers, where the reverse primer contained a 6-bp error-correcting barcode unique to each sample (515 f: 5′-GTGCCAGCMGCCGCGGTAA-3′, 907r: 5′-CCGTCAATTCCTTTGAGTTT-3′). Amplification was performed using an initial denaturation at 98 °C for 1 min followed by 30 cycles of denaturation at 98 °C for 10 s, annealing at 50 °C for 30 s, elongation at 72 °C for 30 s, and a final step of 72 °C for 5 min. All PCR reactions were carried out using Phusion® High-Fidelity PCR Master Mix (NEB, Ipswich, Massachusetts, USA). PCR products were run in an electrophoresis chamber on a 2% agarose gel to confirm the successful amplification of the target gene. DNA bands of 400–450 bp, corresponding to the 16S rRNA amplicon, were excised and purified using the GeneJET Gel Extraction Kit (Thermo Scientific, USA) according to the manufacturer’s instructions. Purified amplicons were used for library preparation and pyrosequencing. Sequencing libraries were generated using NEB Next® Ultra™ DNA Library Prep Kit for Illumina (NEB, Ipswich, Massachusetts, USA), following the manufacturer’s recommendations, and index codes were added. A Qubit@ 2.0 Fluorometer (Thermo Scientific, Waltham, Massachusetts, USA) and Agilent Bioanalyzer 2100 system (Agilent, Santa Clara, California, USA) were used to assess the quality of the library. Pyrosequencing was performed on the Illumina HiSeq. 2 × 250 platform (Illumina, San Diego, California, USA) according to the manufacturer’s instructions.

### Bioinformatics and statistical analysis

Sequencing reads were assigned to each sample based on unique barcodes, and truncated by cutting off the barcode and primer sequence. The original DNA fragments were merged into tags using FLASH (v1.2.7)^49^. Quality filtering of the raw tags was performed under specific filtering conditions to generate high-quality clean tags according to the QIIME (v1.7.0) quality controlled process^50^. In order to generate effective tags, the chimeric sequences were removed from the clean tags using the UCHIME algorithm^51^ based on the reference database (Gold database)^52^. After selecting representative species for each operational taxonomic unit (OTU), each of the remaining sequences was assigned to an OTU when at least 97% threshold identity was obtained using UPARSE software (v7.0.1001)^53^. The taxonomy of each OTU representative sequence was assigned for further annotation using the Ribosomal Database Project (RDP) classifier (v2.2)^54^ based on the GreenGenes Database^55^. Subsequently, the information pertaining to OTU abundance was normalized using a standard sequence number corresponding to the sample with the least number of sequences. Subsequent analyses were performed based on the normalized data. Alpha diversity analysis is usually used to study the complexity of species diversity in samples^56^. In this study, we evaluated the alpha diversity of the samples using the Chao1 and Shannon indices^57,58^, which were calculated by QIIME (v1.7.0)^50^. Moreover, we evaluated the sample size and the sequencing depth using Good’s coverage index^59^. Beta diversity analysis was used to evaluate the differences in microbial community composition in the samples^60^. In this study, the weighted UniFrac distance matrixes were calculated by QIIME (v1.7.0) to evaluate the differences in the microbiotal community compositions of all the test samples from the three gut locations^61^, and the results were visualized by PCoA and dendrograms using R software (v2.15.3) and FastTree (v1.9.0)^62^, respectively. Finally, in order to evaluate the differences in phylogenetic composition between the groups, multi-response permutation procedure (MRPP) analysis was performed.

Linear discriminant analysis coupled with the effect size (LEfSe) algorithm was used to identify the OTUs that differed significantly among the three groups (ileum, cecum, and colon) based on the OTU relative abundance values^63^. Briefly, the algorithm first used the non-parametric factorial Kruskal-Wallis (KW) sum-rank test to detect the taxa with significantly different abundances, followed by pairwise Wilcoxon tests to detect biological consistency between the two groups. Finally, an LDA score was used to estimate the effect size of each differentially abundant feature. The functional capacity of the gut microbiome was estimated by inferring metabolic functionality from the 16S rRNA gene sequencing data using the Phylogenetic Investigation of Communities by Reconstruction of Unobserved States (PICRUSt) v1.0 software^21^. Z-scores were calculated to construct a heatmap to demonstrate the relative abundance of the pathways in each group with the formula z = (x − μ)/σ, where x is the relative abundance of the pathways in each group, μ is the mean value of the relative abundances of the pathways in all groups, and σ is the standard deviation of the relative abundances. The Wilcoxon Signed-Ranks test was also used to determine the significance of the OTU number, alpha diversity, gene pathways, and OTU relative abundance between the sample groups.

### Data availability

All 16S rRNA gene sequencing data were submitted to the NCBI’s Sequence Read Archive (SRA) database under the accession ID PRJNA391047.

## Electronic supplementary material

Supplementary Figures and Tables
