# Supplementary material for: A global comparison of the microbiome compositions of three gut locations in commercial pigs with extreme feed conversion ratios
Source: Sci Rep. 2018 Mar 14;8:4536. doi: 10.1038/s41598-018-22692-0 (PMC5852056; doi:10.1038/s41598-018-22692-0)
Supplement: Supplementary file 1 — Supplementary Figures and Tables [file 41598_2018_22692_MOESM1_ESM.doc]

**Supplementary Information of**

**A global comparison of the microbiome compositions of three gut locations in commercial pigs with extreme feed conversion ratios**

Jianping Quan ^1,$^, Gengyuan Cai^1,2,$^, Jian Ye^1^, Ming Yang^2^, Rongrong Ding ^1^, Xingwang Wang^1^, Enqin Zheng^1^, Disheng Fu^1^, Shaoyun Li^1^, Shenping Zhou^1^,Dewu Liu^1^, Jie Yang^1,*^ , Zhenfang Wu^1,2,*^

^1^College of Animal Science and National Engineering Research Center for Breeding Swine Industry, South China Agricultural University, Guangdong, P.R. China.

^2^National Engineering Research Center for Breeding Swine Industry, Guangdong Wens Foodstuffs Co., Ltd, Guangdong, P.R. China.

^$^These authors contributed equally to this work

^*^Correspondence and requests for materials should be addressed to J.Y. (email: [jieyang2012@hotmail.com](mailto:jieyang2012@hotmail.com)) or Z.W. (email: [wzfeamil@163.com](mailto:wzfeamil@163.com))

**Supplementary Figures**

**
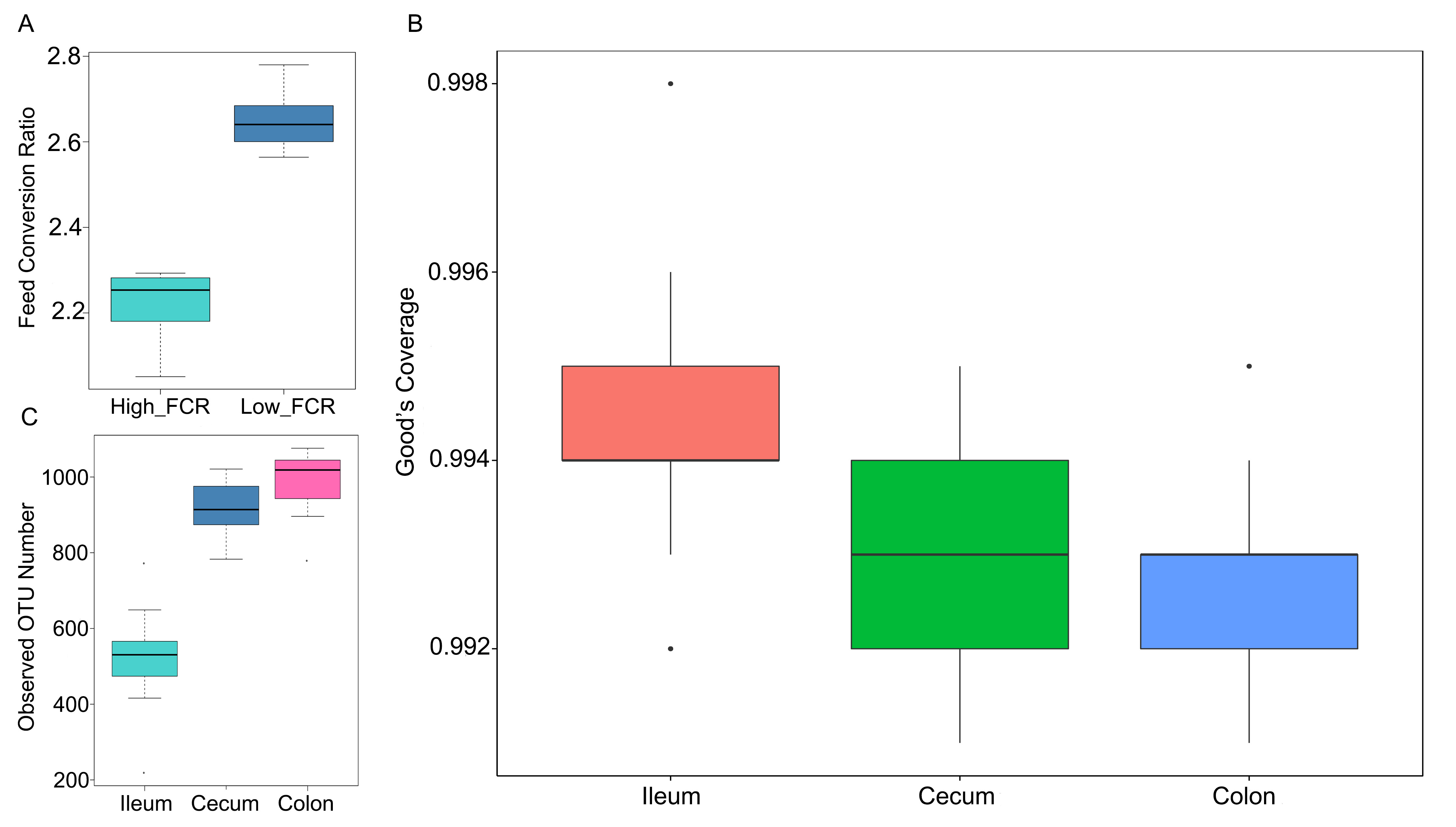
**

**Supplementary Figure S1**

A. The average feed conversion rate (FCR) of high and low FCR groups.

B. The Good’s coverage index of the samples in the three gut locations.

C**.** The average OTU numbers of the samples in the three gut locations.

**
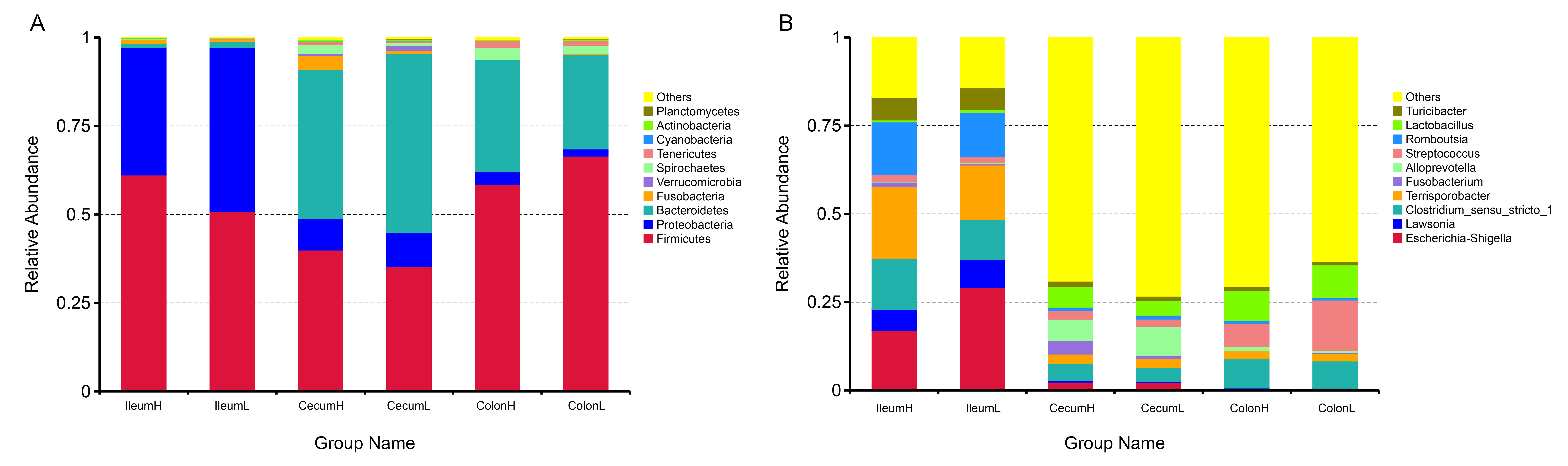
**

**Supplementary Figure S2**

A. Microbial composition at the phylum level for high and low FCR groups in each gut location.

B. Microbial composition at the genus level for high and low FCR groups at each gut location.

**Supplementary Tables**

**Table S1. The MRPP analysis of between-group and within-group distances.**

| Group | A^1^ | Observed-delta | Expected-delta | Significance |
| --- | --- | --- | --- | --- |
| Cecum-Colon | 0.0994 | 0.4788 | 0.5317 | 0.001 |
| Cecum-Ileum | 0.2127 | 0.5553 | 0.7054 | 0.001 |
| Colon-Ileum | 0.2730 | 0.4944 | 0.6800 | 0.001 |

1, The A value over than 0 reveals that inter-group difference bigger than the intra-group difference, smaller Observe-delta value reflects the smaller intra-group difference, bigger Expected-delta value reflect the bigger inter-group difference.

**Table S2.** **The relative abundance of the top 10 phyla.**

| Taxonomy | Ileum | Cecum | Colon |
| --- | --- | --- | --- |
| *Firmicutes* | 56.011 | 37.704 | 62.521 |
| *Proteobacteria* | 41.218 | 9.268 | 2.781 |
| *Bacteroidetes* | 1.342 | 46.347 | 29.203 |
| *Fusobacteria* | 0.926 | 2.284 | 0.100 |
| *Verrucomicrobia* | 0.0073 | 1.060 | 0.075 |
| *Spirochaetes* | 0.126 | 1.716 | 2.797 |
| *Tenericutes* | 0.132 | 0.593 | 1.649 |
| *Cyanobacteria* | 0.039 | 0.177 | 0.0664 |
| *Cyanobacteria* | 0.0888 | 0.344 | 0.213 |
| *Planctomycetes* | 0.0093 | 0.081 | 0.167 |

**Table S3.** **The relative abundance of the top 10 genera.**

| Taxonomy | Ileum | Cecum | Colon |
| --- | --- | --- | --- |
| *Escherichia-Shigella* | 23.124 | 2.308 | 0.482 |
| *Lawsonia* | 6.909 | 0.357 | 0.205 |
| *Clostridium sensu stricto1* | 12.869 | 4.376 | 7.976 |
| *Terrisporobacter* | 17.907 | 2.627 | 2.240 |
| *Fusobacterium* | 0.924 | 2.279 | 0.100 |
| *Alloprevotella* | 0.146 | 7.233 | 0.899 |
| *Streptococcus* | 1.848 | 2.207 | 10.377 |
| *Romboutsia* | 13.688 | 1.087 | 0.824 |
| *Lactobacillus* | 0.741 | 5.012 | 8.815 |
| *Turicibacter* | 6.187 | 1.376 | 1.078 |

**Table S4. The differentially bacterial communities among three gut locations using LEfSe at the OTU level**

| OTU ID | Taxon^1^ | | | Relative Abundance | | | LDA Score  (log10) | *P*-value^2^ |
| --- | --- | --- | --- | --- | --- | --- | --- | --- |
|  | Phylum | Family | Genus | Ileum | Cecum | Colon |  |  |
| OUT1 | *Proteobacteria* | *Enterobacteriaceae* | *Escherichia-Shigella* | 22.818 | 2.434 | 0.488 | 5.36 | 3.69E-12 |
| OUT2 | *Firmicutes* | *Peptostreptococcaceae* | *Terrisporobacter* | 18.467 | 2.611 | 2.265 | 5.25 | 5.18E-10 |
| OUT4 | *Firmicutes* | *Peptostreptococcaceae* | *Romboutsia* | 13.896 | 1.084 | 0.840 | 5.14 | 1.69E-11 |
| OTU8 | *Proteobacteria* | *Desulfovibrionaceae* | *Lawsonia* | 6.089 | 0.362 | 0.191 | 4.84 | 4.81E-06 |
| OTU21 | *Proteobacteria* | *Pasteurellaceae* | *Actinobacillus* | 3.544 | 0.229 | 0.074 | 4.55 | 1.05E-10 |
| OUT3 | *Firmicutes* | *Streptococcaceae* | *Streptococcus* | 1.357 | 2.093 | 9.926 | 5.01 | 4.05E-10 |
| OUT5 | *Firmicutes* | *Clostridiaceae* | *Clostridium sensu stricto1* | 5.640 | 2.769 | 6.093 | 4.78 | 0.00082 |
| OUT6 | *Firmicutes* | *Lactobacillaceae* | *Lactobacillus* | 0.387 | 3.145 | 5.543 | 4.76 | 3.25E-10 |
| OUT15 | *Firmicutes* | *Christensenellaceae* | - | 0.052 | 0.176 | 2.643 | 4.43 | 3.22E-11 |
| OUT18 | *Firmicutes* | *Lactobacillaceae* | *Lactobacillus* | 0.139 | 1.244 | 2.196 | 4.35 | 1.34E-11 |
| OUT10 | *Proteobacteria* | *Neisseriaceae* | *Leeia* | 0.109 | 3.087 | 0.132 | 4.48 | 3.41E-10 |
| OUT28 | *Bacteroidetes* | *Prevotellaceae* | *Prevotella* | 0.030 | 3.016 | 0.714 | 4.46 | 6.44E-12 |
| OUT63 | *Bacteroidetes* | *Prevotellaceae* | *Alloprevotella* | 0.053 | 2.252 | 0.299 | 4.36 | 7.09E-12 |

1, The OTUs were annotation at phylum, family and genus level, (-): the OTU cannot be further classification at this annotation level

2, *P*-value < 0.05 was considered as significant differences.

**Table S5.** **The relative abundance of functional pathways** microbiota among three gut locations.

| **KEGG Description** | **Ileum** | **Cecum** | **Colon** |
| --- | --- | --- | --- |
| Neurodegenerative diseases | 0.154^a^ | 0.129^b^ | 0.103^c^ |
| Enzyme families | 2.810^a^ | 2.451^b^ | 2.411^b^ |
| Environmental adaptation | 0.168^a^ | 0.158^b^ | 0.158^b^ |
| Cellular processes and signaling | 4.544^a^ | 3.993^b^ | 3.743^b^ |
| Infectious diseases | 0.454^a^ | 0.398^b^ | 0.388^b^ |
| Poorly characterized  Membrane transport | 5.078^a^  15.024^a^ | 4.869^b^  11.039^b^ | 4.815^b^  11.979^c^ |
| Cell motility | 3.740^a^ | 2.286^b^ | 2.505^c^ |
| Signal transduction | 1.983^a^ | 1.523^b^ | 1.582^b^ |
| Xenobiotics biodegradation and metabolism | 1.804^a^ | 1.562^b^ | 1.673^c^ |
| Transcription | 2.922^a^ | 2.620^b^ | 2.840^c^ |
| Metabolism of other amino acids | 1.501^a^ | 1.552^b^ | 1.478^c^ |
| Signaling molecules and interaction  Metabolism of cofactors and vitamins | 0.182^a^  4.054^a^ | 0.185^b^  4.424^b^ | 0.182^a^  4.150^a^ |
| Cancers  Metabolism | 0.100^a^  2.192^a^ | 0.107^b^  2.229^b^ | 0.101^a^  2.194^a^ |
| Digestive system | 0.014^a^ | 0.061^b^ | 0.041^c^ |
| Metabolism of terpenoids and polyketides | 1.581^a^ | 1.709^b^ | 1.658^c^ |
| Glycan biosynthesis and metabolism | 1.905^a^ | 2.664^b^ | 2.226^c^ |
| Folding sorting and degradation | 2.314^a^ | 2.590^b^ | 2.443^c^ |
| Immune system  Carbohydrate metabolism | 0.078^a^  10.224^a^ | 0.091^b^  10.054^b^ | 0.084^c^  10.261^c^ |
| Lipid metabolism | 2.598^a^ | 2.640^b^ | 2.739^c^ |

Superscripts a,b,c indicate for significant differences at *P* < 0.05.

**Table S6. The differentially bacterial communities between high and low FCR pigs among three gut locations using LEfSe at the OTU level**

| Gut Location | OUT ID | Taxon^1^ | | | Relative Abundance | | LDA score  (log 10) | *P*-value^2^ |
| --- | --- | --- | --- | --- | --- | --- | --- | --- |
|  |  | phylum | family | genus | High FCR | Low FCR |  |  |
| Ileum | OTU3 | *Firmicutes* | *Streptococcaceae* | *Streptococcus* | 1.57 | 1.07 | 3.80 | 0.0111 |
| Ileum | OTU367 | *Firmicutes* | *Ruminococcaceae* | *Ruminococcaceae UCG-010* | 0.00232 | 0.000332 | 2.30 | 0.0443 |
| Ileum | OTU437 | *Tenericutes* | *-* | *-* | 0.00266 | 0.000664 | 2.21 | 0.0285 |
| Ileum | OTU205 | *SHA-109* | *-* | *-* | 0.0136 | 0.00498 | 2.13 | 0.0078 |
| Ileum | OTU132 | *Firmicutes* | *Christensenellaceae* | *Christensenellaceae R-7 group* | 0.00498 | 0.000996 | 2.02 | 0.0176 |
| Ileum | OTU1347 | *Firmicutes* | *Lachnospiraceae* | *Roseburia* | 0 | 0.00166 | 2.18 | 0.0329 |
| Ileum | OTU118 | *Bacteroidetes* | *Prevotellaceae* | *Prevotellaceae NK3B31 group* | 0.00365 | 0.0123 | 2.16 | 0.0194 |
| Ileum | OTU997 | *Firmicutes* | *Lachnospiraceae* | *-* | 0 | 0.00299 | 2.15 | 0.0329 |
| Ileum | OTU569 | *Firmicutes* | *Lachnospiraceae* | *-* | 0 | 0.00166 | 2.04 | 0.0329 |
| Ileum | OTU286 | *Bacteroidetes* | *Prevotellaceae* | *-* | 0 | 0.00399 | 2.03 | 0.0329 |
| Ileum | OTU164 | *Bacteroidetes* | *Rikenellaceae* | *Rikenellaceae RC9 gut group* | 0.00299 | 0.00897 | 2.00 | 0.0207 |
| Cecum | OTU456 | *Firmicutes* | *Lachnospiraceae* | *Eubacterium hallii group* | 1.56 | 0.101 | 2.04 | 0.0247 |
| Cecum | OTU1742 | *Firmicutes* | *Clostridiaceae* | *Clostridium sensu stricto1* | 0.933 | 0.356 | 2.1 | 0.0336 |
| Cecum | OTU359 | *Firmicutes* | *Lachnospiraceae* | *Lachnospiraceae UCG 003* | 0.432 | 0.152 | 2.11 | 0.0208 |
| Cecum | OTU172 | *Firmicutes* | *Veillonellaceae* | *Dialister* | 0.298 | 0.0601 | 2.13 | 0.0235 |
| Cecum | OTU166 | *Bacteroidetes* | *Rikenellaceae* | *Rikenellaceae RC9 gut group* | 0.268 | 0.107 | 2.14 | 0.0377 |
| Cecum | OTU248 | *Bacteroidetes* | *Porphyromonadaceae* | *-* | 0.183 | 0.0319 | 2.14 | 0.0463 |
| Cecum | OTU1605 | *Firmicutes* | *Lachnospiraceae* |  | 0.230 | 0.0834 | 2.14 | 0.0262 |
| Cecum | OTU410 | *Bacteroidetes* | *Prevotellaceae* | *Prevotellaceae NK3B31 group* | 0.312 | 0.165 | 2.14 | 0.0447 |
| Cecum | OTU209 | *Lentisphaerae* | *-* | *-* | 0.158 | 0.0402 | 2.17 | 0.0327 |
| Cecum | OTU169 | *Firmicutes* | *Ruminococcaceae* | *Ruminiclostridium* | 0.251 | 0.138 | 2.17 | 0.0282 |
| Cecum | OTU290 | *Firmicutes* | *Lachnospiraceae* | *-* | 0.191 | 0.0751 | 2.16 | 0.0377 |
| Cecum | OTU141 | *Firmicutes* | *Lachnospiraceae* | *Lachnospiraceae NK4A136 group* | 0.204 | 0.088 | 2.18 | 0.0209 |
| Cecum | OTU205 | *SHA-109* | *-* | *-* | 0.133 | 0.0395 | 2.13 | 0.0078 |
| Cecum | OTU1431 | *Firmicutes* | *Lachnospiraceae* | *-* | 0.244 | 0.137 | 2.25 | 0.0111 |
| Cecum | OTU1735 | *Bacteroidetes* | *Prevotellaceae* | *Prevotellaceae NK3B31 group* | 0.121 | 0.0408 | 2.25 | 0.0325 |
| Cecum | OTU267 | *Tenericutes* | *-* | *-* | 0.167 | 0.0687 | 2.29 | 0.0208 |
| Cecum | OTU1525 | *Bacteroidetes* | *Rikenellaceae* | *Rikenellaceae RC9 gut group* | 0.158 | 0.0737 | 2.3 | 0.0433 |
| Cecum | OTU108 | *Spirochaetes* | *Spirochaetaceae* | *Treponema* | 0.103 | 0.0176 | 2.38 | 0.0323 |
| Cecum | OTU206 | *Firmicutes* | *Ruminococcaceae* | *Ruminococcus* | 0.133 | 0.0352 | 2.42 | 0.0111 |
| Cecum | OTU120 | *Bacteroidetes* | *Bacteroidales S24-7 group* | *-* | 0.143 | 0.0528 | 2.47 | 0.0303 |
| Cecum | OTU1017 | *Firmicutes* | *Ruminococcaceae* | *Ruminiclostridium* | 0.131 | 0.0565 | 2.48 | 0.0111 |
| Cecum | OTU243 | *Bacteroidetes* | *Bacteroidales RF16 group* | *-* | 0.130 | 0.0312 | 2.58 | 0.0326 |
| Cecum | OTU220 | *Firmicutes* | *Lachnospiraceae* | *Lachnospiraceae NK4B4 group* | 0.0893 | 0.0223 | 2.59 | 0.0282 |
| Cecum | OTU104 | *Firmicutes* | *Lachnospiraceae* | *-* | 0.0658 | 0.0163 | 2.63 | 0.0111 |
| Cecum | OTU187 | *Spirochaetes* | *Spirochaetaceae* | *Treponema* | 0.0970 | 0.0399 | 2.63 | 0.0067 |
| Cecum | OTU296 | *Firmicutes* | *Lachnospiraceae* | *Lachnospiraceae NK4A136 group* | 0.0684 | 0.0179 | 2.64 | 0.0178 |
| Cecum | OTU76 | *Bacteroidetes* | *Rikenellaceae* | *Rikenellaceae RC9 gut group* | 0.134 | 0.0933 | 2.64 | 0.0282 |
| Cecum | OTU123 | *Firmicutes* | *Lachnospiraceae* | *-* | 0.0535 | 0.0169 | 2.66 | 0.013 |
| Cecum | OTU228 | *Planctomycetes* | *Planctomycetaceae* | *p-1088-a5 gut group* | 0.0638 | 0.0186 | 2.67 | 0.0209 |
| Cecum | OTU77 | *Bacteroidetes* | *Rikenellaceae* | *Rikenellaceae RC9 gut group* | 0.0638 | 0.0242 | 2.69 | 0.0243 |
| Cecum | OTU174 | *Firmicutes* | *Ruminococcaceae* | *Ruminococcus* | 0.0581 | 0.0319 | 2.71 | 0.0022 |
| Cecum | OTU100 | *Firmicutes* | *Ruminococcaceae* | *Ruminococcus* | 0.0727 | 0.0359 | 2.73 | 0.0377 |
| Cecum | OTU119 | *Firmicutes* | *Lachnospiraceae* | *-* | 0.0462 | 0.0229 | 2.75 | 0.013 |
| Cecum | OTU107 | *Bacteroidetes* | *Rikenellaceae* | *Rikenellaceae RC9 gut group* | 0.0415 | 0.0133 | 2.76 | 0.0377 |
| Cecum | OTU1020 | *Bacteroidetes* | *Porphyromonadaceae* | *-* | 0.0591 | 0.0339 | 2.79 | 0.0153 |
| Cecum | OTU52 | *Firmicutes* | *Lachnospiraceae* | *-* | 0.0315 | 0.00531 | 2.82 | 0.0327 |
| Cecum | OTU49 | *Firmicutes* | *Lachnospiraceae* | *Lachnospiraceae XPB1014 group* | 0.0405 | 0.0153 | 2.84 | 0.0022 |
| Cecum | OTU190 | *Bacteroidetes* | *Bacteroidales S24-7 group* | *-* | 0.0418 | 0.0153 | 2.86 | 0.0179 |
| Cecum | OTU75 | *Bacteroidetes* | *Porphyromonadaceae* | *-* | 0.0621 | 0.0382 | 2.94 | 0.0377 |
| Cecum | OTU127 | *Spirochaetes* | *Spirochaetaceae* | *Treponema* | 0.0306 | 0.00863 | 3.01 | 0.0327 |
| Cecum | OTU44 | *Bacteroidetes* | *Rikenellaceae* | *Rikenellaceae RC9 gut group* | 0.0369 | 0.0106 | 3.14 | 0.0153 |
| Cecum | OTU16 | *Bacteroidetes* | *Prevotellaceae* | *Prevotellaceae UCG 001* | 0.0186 | 0.00199 | 3.48 | 0.0047 |
| Cecum | OTU14 | *Bacteroidetes* | *Rikenellaceae* | *Rikenellaceae RC9 gut group* | 0.0189 | 0.00266 | 3.95 | 0.0079 |
| Cecum | OTU33 | *Firmicutes* | *Erysipelotrichaceae* | *-* | 0.375 | 0.918 | 3.51 | 0.0327 |
| Cecum | OTU51 | *Bacteroidetes* | *Bacteroidaceae* | *Bacteroides* | 0.0325 | 1.06 | 3.51 | 0.0071 |
| Cecum | OTU62 | *Bacteroidetes* | *Porphyromonadaceae* | *Parabacteroides* | 0.706 | 1.21 | 3.39 | 0.0377 |
| Cecum | OTU161 | *Firmicutes* | *Ruminococcaceae* | *Eubacterium coprostanoligenes group* | 0.0698 | 0.271 | 3.01 | 0.0094 |
| Cecum | OTU162 | *Bacteroidetes* | *-* | *-* | 0.0704 | 0.259 | 2.99 | 0.0282 |
| Cecum | OTU194 | *Bacteroidetes* | *Bacteroidaceae* | *Bacteroides* | 0.0273 | 0.194 | 2.77 | 0.0354 |
| Cecum | OTU346 | *Bacteroidetes* | *Bacteroidaceae* | *Bacteroides* | 0.00431 | 0.183 | 2.76 | 0.0152 |
| Cecum | OTU180 | *Proteobacteria* | *Alcaligenaceae* | *Sutterella* | 0.0571 | 0.158 | 2.73 | 0.0209 |
| Cecum | OTU255 | *Bacteroidetes* | *Bacteroidaceae* | *Bacteroides* | 0.00166 | 0.0910 | 2.51 | 0.0009 |
| Cecum | OTU195 | *Firmicutes* | *Lachnospiraceae* | *Lachnoclostridium* | 0.0315 | 0.077 | 2.35 | 0.013 |
| Cecum | OTU294 | *Proteobacteria* | *Alcaligenaceae* | *Sutterella* | 0.0146 | 0.0494 | 2.29 | 0.0039 |
| Cecum | OTU268 | *Firmicutes* | *Ruminococcaceae* | *-* | 0.0276 | 0.0684 | 2.28 | 0.0282 |
| Colon | OTU273 | *Firmicutes* | *Ruminococcaceae* | *Ruminococcaceae UCG 010* | 0.0369 | 0.0206 | 2.22 | 0.0377 |
| Colon | OTU474 | *Tenericutes* | *-* | *-* | 0.0106 | 0.00531 | 2.26 | 0.0288 |
| Colon | OTU414 | *Firmicutes* | *Ruminococcaceae* | *Ruminococcaceae UCG 010* | 0.0232 | 0.0083 | 2.35 | 0.0325 |
| Colon | OTU321 | *Actinobacteria* | *Coriobacteriaceae* | *-* | 0.0199 | 0.0116 | 2.36 | 0.0431 |
| Colon | OTU397 | *Firmicutes* | *Clostridiales vadinBB60 group* | *-* | 0.0289 | 0.00731 | 2.22 | 0.0107 |
| Colon | OTU652 | *Firmicutes* | *-* | *-* | 0.00863 | 0.000996 | 2.33 | 0.0068 |
| Colon | OTU661 | *Tenericutes* | *-* | *-* | 0.0408 | 0.0193 | 2.21 | 0.0209 |
| Colon | OTU651 | *Firmicutes* | *Ruminococcaceae* | *Ruminococcaceae UCG 010* | 0.00631 | 0.00133 | 2.27 | 0.0336 |
| Colon | OTU295 | *Bacteroidetes* | *Prevotellaceae* |  | 0.0259 | 0.00897 | 2.31 | 0.0492 |
| Colon | OTU608 | *Bacteroidetes* | *Prevotellaceae* | *Prevotella* | 0.0232 | 0.000996 | 2.4 | 0.0168 |
| Colon | OTU760 | *Firmicutes* | *Ruminococcaceae* | *Ruminococcaceae UCG 010* | 0.00897 | 0.00465 | 2.45 | 0.0368 |
| Colon | OTU205 | *SHA-109* | *-* | *-* | 0.0598 | 0.0339 | 2.13 | 0.0078 |
| Colon | OTU1385 | *Firmicutes* | *Ruminococcaceae* | *Ruminococcaceae UCG 014* | 0.00299 | 0.000332 | 2.51 | 0.0443 |
| Colon | OTU896 | *Tenericutes* | *-* | *-* | 0.0216 | 0.0103 | 2.55 | 0.0281 |
| Colon | OTU212 | *Bacteroidetes* | *Rikenellaceae* | *Rikenellaceae RC9 gut group* | 0.108 | 0.0588 | 2.48 | 0.0153 |
| Colon | OTU666 | *Firmicutes* | *Ruminococcaceae* | *Anaerotruncus* | 0.00513 | 0.00133 | 2.61 | 0.0207 |
| Colon | OTU928 | *Firmicutes* | *Ruminococcaceae* | *Eubacterium coprostanoligenes group* | 0.00465 | 0.000332 | 2.54 | 0.0081 |
| Colon | OTU914 | *Firmicutes* | *Ruminococcaceae* | *Eubacterium coprostanoligenes* | 0.0083 | 0.00166 | 2.53 | 0.0052 |
| Colon | OTU166 | *Bacteroidetes* | *Rikenellaceae* | *Rikenellaceae RC9 gut group* | 0.128 | 0.0667 | 2.14 | 0.0377 |
| Colon | OTU1060 | *Firmicutes* | *Ruminococcaceae* | *-* | 0.002657 | 0.000664 | 2.65 | 0.0442 |
| Colon | OTU1650 | *Tenericutes* | *-* | *-* | 0.005313 | 0 | 2.55 | 0.0147 |
| Colon | OTU569 | *Firmicutes* | *Lachnospiraceae* | *-* | 0.00897 | 0.00332 | 2.04 | 0.0329 |
| Colon | OTU938 | *Firmicutes* | *-* | *-* | 0.00266 | 0 | 2.97 | 0.0025 |
| Colon | OTU76 | *Bacteroidetes* | *Rikenellaceae* | *Rikenellaceae RC9 gut group* | 0.315 | 0 | 2.64 | 0.0282 |
| Colon | OTU1355 | *Proteobacteria* | *Desulfovibrionaceae* | *Lawsonia* | 0.00133 | 0 | 2.88 | 0.0329 |
| Colon | OTU1308 | *Firmicutes* | *Clostridiales vadinBB60 group* | *-* | 0.00199 | 0 | 2.64 | 0.0329 |
| Colon | OTU685 | *Firmicutes* | *Erysipelotrichaceae* | *Dielma* | 0.00299 | 0 | 2.81 | 0.0147 |
| Colon | OTU319 | *Spirochaetes* | *Spirochaetaceae* | *Treponema* | 0.101 | 0.0183 | 2.64 | 0.0496 |
| Colon | OTU739 | *Firmicutes* | *Ruminococcaceae* | *Ruminococcaceae UCG 010* | 0.00166 | 0 | 2.91 | 0.0329 |
| Colon | OTU1128 | *Firmicutes* | *Ruminococcaceae* | *Hydrogenoanaerobacterium* | 0.00465 | 0.000996 | 2.88 | 0.0105 |
| Colon | OTU1202 | *Firmicutes* | *Clostridiales vadinBB60 group* | *-* | 0.00133 | 0 | 3.21 | 0.0329 |
| Colon | OTU723 | *Firmicutes* | *Ruminococcaceae* | *Ruminiclostridium* | 0.0123 | 0.00266 | 2.69 | 0.002 |
| Colon | OTU77 | *Bacteroidetes* | *Rikenellaceae* | *Rikenellaceae RC9 gut group* | 0.241 | 0.132 | 2.69 | 0.0243 |
| Colon | OTU832 | *Firmicutes* | *-* | *-* | 0.00133 | 0 | 2.92 | 0.0329 |
| Colon | OTU996 | *Proteobacteria* | *-* | *-* | 0.00266 | 0.000332 | 3.05 | 0.0212 |
| Colon | OTU98 | *Firmicutes* | *Ruminococcaceae* | *Ruminococcus* | 0.297 | 0.11 | 3.08 | 0.0243 |
| Colon | OTU44 | *Bacteroidetes* | *Rikenellaceae* | *Rikenellaceae RC9 gut group* | 0.551 | 0.275 | 3.14 | 0.0153 |
| Colon | OTU177 | *Spirochaetes* | *Spirochaetaceae* | *Treponema* | 0.538 | 0.161 | 3.3 | 0.0433 |
| Colon | OTU3 | *Firmicutes* | *Streptococcaceae* | *Streptococcus* | 6.32 | 14.1 | 3.8 | 0.0111 |
| Colon | OTU1032 | *Firmicutes* | *Ruminococcaceae* | *-* | 0 | 0.00199 | 3.33 | 0.0147 |
| Colon | OTU47 | *Firmicutes* | *Lachnospiraceae* | *Lachnospiraceae AC2044 group* | 0.29 | 0.487 | 3.03 | 0.0377 |
| Colon | OTU380 | *Firmicutes* | *Staphylococcaceae* | *Macrococcus* | 0.000664 | 0.00465 | 2.86 | 0.0169 |
| Colon | OTU543 | *Firmicutes* | *Ruminococcaceae* | *-* | 0.000664 | 0.00399 | 2.83 | 0.0202 |
| Colon | OTU162 | *Bacteroidetes* | *-* | *-* | 0.0445 | 0.101 | 2.99 | 0.0282 |
| Colon | OTU1572 | *Firmicutes* | *Ruminococcaceae* | *-* | 0.161 | 0.217 | 2.52 | 0.0433 |
| Colon | OTU210 | *Bacteroidetes* | *Bacteroidales S24-7 group* | *-* | 0.0379 | 0.0814 | 2.46 | 0.0282 |
| Colon | OTU1628 | *Firmicutes* | *Streptococcaceae* | *Streptococcus* | 0.0223 | 0.0369 | 2.51 | 0.0326 |
| Colon | OTU978 | *Firmicutes* | *Lachnospiraceae* | *-* | 0.00531 | 0.00897 | 2.51 | 0.0185 |
| Colon | OTU509 | *Firmicutes* | *Ruminococcaceae* | *-* | 0.00199 | 0.00731 | 2.45 | 0.0128 |
| Colon | OTU875 | *Firmicutes* | *Streptococcaceae* | *Streptococcus* | 0.0226 | 0.0478 | 2.4 | 0.0433 |
| Colon | OTU194 | *Bacteroidetes* | *Bacteroidaceae* | *Bacteroides* | 0.00199 | 0.0153 | 2.77 | 0.0354 |
| Colon | OTU533 | *Firmicutes* | *Ruminococcaceae* | *Ruminiclostridium* | 0.00513 | 0.0123 | 2.52 | 0.0424 |
| Colon | OTU1432 | *Firmicutes* | *Streptococcaceae* | *Streptococcus* | 0.00598 | 0.0236 | 2.44 | 0.0488 |
| Colon | OTU814 | *Bacteroidetes* | *Bacteroidales S24-7 group* | *-* | 0.00797 | 0.0179 | 2.49 | 0.0149 |
| Colon | OTU266 | *Firmicutes* | *Prevotellaceae* | *Prevotellaceae UCG 003* | 0.0112 | 0.0113 | 2.16 | 0.0438 |

1, The OTUs were annotation at phylum, family and genus level; (-): the OTU cannot be further classification at this annotation level

2, *P*-value < 0.05 was considered as significant differences.

**Table S7. The relative abundance of functional pathways microbiota between high and low FCR groups in the** three gut locations.

| **Gut Location** | **KEGG Description** | **High FCR** | **Low FCR** | **Significance^1^** |
| --- | --- | --- | --- | --- |
| Ileum | Neurodegenerative Diseases | 0.146 | 0.162 | ** |
|  | Poorly Characterized | 5.02 | 5.13 | * |
|  | Infectious Diseases | 0.444 | 0.465 | *** |
| Cecum | Carbohydrate Metabolism | 10.11 | 9.99 | ** |
|  | Lipid Metabolism | 2.69 | 2.60 | * |
| Colon | Metabolism of other amino acids | 1.57 | 1.46 | ** |
|  | Cancers | 0.104 | 0.098 | * |
|  | Signaling molecules and interaction | 0.185 | 0.178 | * |
|  | Metabolism of cofactors and vitamins | 4.19 | 4.11 | * |
|  | Digestive system | 0.041 | 0.034 | * |
|  | Glycan biosynthesis and metabolism | 2.26 | 2.19 | * |
|  | Folding Sorting and Degradation | 2.45 | 2.38 | * |
|  | Immune System | 0.086 | 0.080 | * |
|  | Metabolism of Terpenoids and Polyketides | 1.67 | 1.60 | * |

1, * indicate for significant differences at *P* < 0.05, ** indicate for significant differences at *P* < 0.01, *** indicate for significant differences at *P* < 0.001.
